# Identifying health and healthcare priorities in rural areas: A concept mapping study informed by consumers, health professionals and researchers

**DOI:** 10.1186/s12961-024-01163-1

**Published:** 2024-08-13

**Authors:** Anna Wong Shee, Alex Donaldson, Renee P. Clapham, John C. Aitken, Jaithri Ananthapavan, Anna Ugalde, Vincent L. Versace, Kevin Mc Namara

**Affiliations:** 1 Grampians Health, 102 Ascot St Sth, Ballarat, VIC 3350 Australia; 2 Deakin Rural Health, Deakin University, Warrnambool, Australia; 3 La Trobe University, Bundoora, Australia; 4 Ballarat Health Services, Ballarat, Australia; 5 Rural Northwest Health, Warracknabeal, Australia; 6 Deakin University, Burwood, Australia

**Keywords:** Access, Healthcare, Health priorities, Health service, Priority-setting, Public values, Public engagement, Rural, Regional

## Abstract

**Background:**

It is vital that health service delivery and health interventions address patients’ needs or preferences, are relevant for practice and can be implemented. Involving those who will use or deliver healthcare in priority-setting can lead to health service delivery and research that is more meaningful and impactful. This is particularly crucial in rural communities, where limited resources and disparities in healthcare and health outcomes are often more pronounced. The aim of this study was to determine the health and healthcare priorities in rural communities using a region-wide community engagement approach.

**Methods:**

This multi-methods study was conducted in five rural communities in the Grampians region, Western Victoria, Australia. It involved six concept mapping steps: (1) preparation, (2) generation (brainstorming statements and identifying rating criteria), (3) structuring statements (sorting and rating statements), (4) representation of statements, (5) interpretation of the concept map and (6) utilization. Community forums, surveys and stakeholder consultations with community members and health professionals were used in Step 2. An innovative online group concept mapping platform, involving consumers, health professionals and researchers was used in Step 3.

**Results:**

Overall, 117 community members and 70 health professionals identified 400 health and healthcare issues. Six stakeholder consultation sessions (with 16 community members and 16 health professionals) identified three key values for prioritizing health issues: equal access for equal need, effectiveness and impact (number of people affected). Actionable priorities for healthcare delivery were largely related to access issues, such as the challenges navigating the healthcare system, particularly for people with mental health issues; the lack of sufficient general practitioners and other health providers; the high travel costs; and poor internet coverage often impacting technology-based interventions for people in rural areas.

**Conclusions:**

This study identified actionable health and healthcare priorities from the perspective of healthcare service users and providers in rural communities in Western Victoria. Issues related to access, such as the inequities in healthcare costs, the perceived lack of quality and availability of services, particularly in mental health and disability, were identified as priorities. These insights can guide future research, policy-making and resource allocation efforts to improve healthcare access, quality and equity in rural communities.

**Supplementary Information:**

The online version contains supplementary material available at 10.1186/s12961-024-01163-1.

## Introduction

All healthcare systems have to make choices about how to allocate scarce healthcare resources. There are growing concerns over health service inefficiencies, unnecessary variation in care, rising costs and unmet needs, particularly in rural areas [1]. These issues highlight the need to be explicit about how healthcare services are prioritized [2, 3]. Priority-setting, “the task of determining the priority to be assigned to a service, a service development or an individual patient at a given point in time” [4], is complex and challenging for decision-makers. There is growing recognition of the benefits of public participation in priority-setting including increased public understanding and acceptance of the need to make choices and increased accountability for decision-makers [5]. End-users, those interested in and/or who benefit from improved healthcare delivery (patients, community members and health professionals), can contribute context-specific knowledge to help identify health and healthcare priorities. Their involvement can help ensure healthcare delivery and health interventions are pragmatic, patient-centred and feasible [6]. Public involvement can help identify community values and principles to guide healthcare priority-setting and contributes to a shared ownership in healthcare decision-making [7].

Public involvement in healthcare priority-setting in rural areas has been limited [8]. It is well recognized that people with the same health problems do not always receive the same healthcare or the healthcare they need [9]. There is considerable evidence that where a person lives can determine the health service or health professional they consult and the type of treatment they receive [9, 10]. For example, in Australia, distance to a radiotherapy centre has been shown to be strongly associated with increased rates of mastectomy for early breast cancer [11]. Unnecessary variation in healthcare raises significant equity, safety and cost concerns, particularly in rural communities, which already experience significant health inequities compared with metropolitan areas [12]. In addition, a lack of rural-specific evidence makes it more difficult for policy-makers to make evidence-informed healthcare decisions in rural communities [13]. Given the relative absence of evidence and limited resources in rural compared with metropolitan communities [13], there is perhaps even greater value in engaging rural communities in identifying health and healthcare priorities to optimize the use of limited resources and healthcare delivery in these communities.

Genuine consumer engagement relies on explicit processes for participation. Recommendations for developing effective patient and public involvement in health technology assessment priority-setting include priority-setting capacity and capability building for patients and community members, investigating patient values that should guide priority-setting [14],clear guidance on the information decision-makers need from the public and a feedback loop that acknowledges public input and usefulness in the priority-setting process [15]. However, beyond health technology assessment, there has been limited use of explicit methods to engage the public and patients meaningfully in health research priority-setting in Australia [16], particularly in rural areas.

There are many methods to solicit opinions from end-users, including surveys, public meetings and targeted feedback from specific groups [2, 17]. Often, these methods are time consuming, labour intensive and challenging in terms of coordination and cost. Equally, they are designed to collect data rather than to engage consumers as partners. Concept mapping is a consensus method that uses a structured conceptualization approach to elicit and evaluate multiple options and provide a visual synthesis of multiple stakeholder perspectives on a topic of interest [18]. It has been used successfully by researchers in academic, organizational and community-based settings [19] and in a range of public health areas, including efforts to prevent chronic disease [20] and identify allied health research priorities [21]. The advantages of concept mapping include access to a variety of stakeholders without substantial cost and human resource, and the capacity for stakeholders to participate remotely and at their own convenience [22].

## Methods

The aim of this study was to identify the health and healthcare priority issues in rural communities using a region-wide community engagement approach.

### Design

This multi-methods study involved community forums, surveys and stakeholder consultation sessions and concept mapping.

### Setting

The study was conducted in five rural communities in the Grampians region in Western Victoria, Australia. For this study, the term “rural” refers to areas classified by the Modified Monash Model (MMM) [23] as regional centres (MMM2), large rural towns (MM3), medium rural towns (MMM4) and small rural towns (MMM5). There were no communities classified as MMM 6 (remote communities) or MMM 7 (very remote communities) in the Grampians catchment. This study focused on general priority-setting for healthcare delivery in rural communities in the Grampians region.

### Research team

Our team involved researchers with clinical and health service experience, expertise in community participation (particularly in rural areas), implementation science, economic evaluation and priority-setting and concept mapping in public health.

### Consent

All participants in the community forums, surveys and stakeholder consultation sessions provided written consent. Guardians provided written consent for participants aged < 18 years. Concept mapping participants provided informed consent the first time they registered on the project page hosted on the Concept Systems groupwisdom™ web platform.

### Procedures

**Fig. 1:**
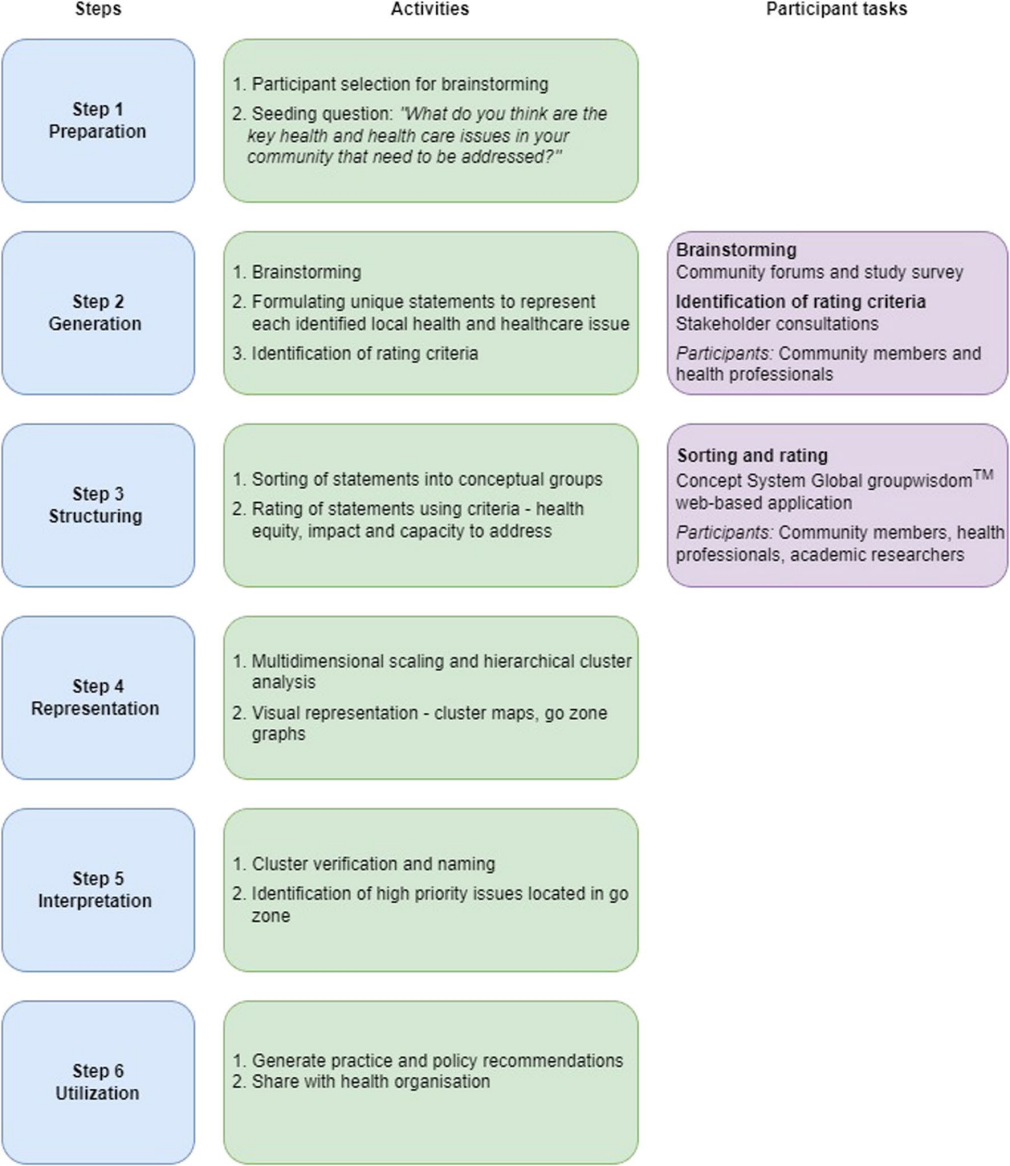
Overview of concept mapping steps (adapted from Kane & Trochim, 2007), activities and participant tasks

An overview of the concept mapping steps, activities and participant tasks is shown in Fig. 1.

The procedures for Steps 1–4 are described below. Steps 5 (interpretation of the concept map) and 6 (implementation) are described in the results and discussion sections, respectively.

### Step 1: preparation – participants and recruitment

Community members and health professionals living or working in one of the five identified rural communities were eligible to participate. Health professionals included allied health, nursing, medical, health promotion professionals and health service managers. Academic researchers could participate if they were based in Victoria (with no geographical restriction) and had expertise in healthcare research, allied health research, health services research or rural health service delivery.

Community members and healthcare professionals were recruited through study flyers distributed to local community organizations (via hard copy), health services (via email) and snowballing in the participating communities. The study was promoted via local media and the official media platforms of the participating health services. People could contact the researcher via phone or email to register their interest in the study. Potential participants could select to participate in the activities in Steps 2 (brainstorming and identification of rating criteria) and 3 (sorting and rating). Participation was voluntary. Refreshments were provided at the face-to-face community forums.

Academic researchers were identified through a combination of personal communication and networks, colleague nomination and snowball sampling. Potential participants were invited to participate via email.

A combination of convenience sampling and purposive sampling approaches were used to maximize the diversity of:

- community member representatives to optimize the inclusion of people with direct experience with the participating health services, including vulnerable groups;
- health disciplines and management representation from the participating health services.

### Step 2: generation – brainstorming

Community forums, surveys and stakeholder consultations were used gain insight into the experiences and perceptions of community members and health professionals of local health issues and service gaps. We aimed to recruit a minimum of 20 community members and 10 health professionals from each of the five rural communities.

#### Community forums

Community forums were held with community members in each participating community between August and December 2019. The purpose was to engage with people living in the Grampians region and to understand their perceptions of the local health and healthcare issues. The community engagement followed a discussion group approach [24] and was used to inform research decision-making, build relationships with patient communities and involve individual patients and health professionals in research. Community forums were held at each study site to provide information about the project and to invite community members to participate in the study. The forums were held at various locations, including neighbourhood houses, agencies providing support to marginalized community members and health services.

We provided definitions of “health” (physical, mental and social well-being) and “health system” issues (that is, health service issues, health professional issues, consumer/patient involvement issues and public health issues). We did not provide information about the health issues of the community (for example, epidemiology, costs, etc.), as we wanted to understand perceptions based on lived experience. The participants brainstormed and discussed their views on the health and healthcare issues they considered important. They were not asked to debate or solve health issues. Participant responses were documented on a whiteboard or on sticky-notes and a member of the research team took field notes during the forum.

#### Survey

A survey was conducted with community members and health professionals to identify the local health issues. The main survey question was a focus statement asking participants “What do you think are the key health and healthcare issues in your community that need to be addressed?” Participants could use their own words to describe as many or as few health and healthcare issues as they wanted. The survey also collected information about respondent’s socioeconomic status, health and well-being and social support. Responses were anonymous. The survey was available both in hardcopy and online.

### Step 2: generation – identification of rating criteria

Stakeholder consultations were conducted between February and July 2020 to identify the criteria that community members and health professionals considered important for determining healthcare priorities in their communities. Separate consultation sessions were held for community members and health professionals to ensure responses were not influenced by actual, potential or perceived power imbalances. Participants who worked or lived in one of the five participating communities and were involved in one consultation session only. Community member and health professional participants from the brainstorming activity in Step 2 were eligible and invited to participate. As the optimal number and size of the stakeholder sessions is not well defined [25], we adopted a pragmatic aim to have at least two community member and two health professional sessions, and a minimum of four and maximum of eight participants per session.

Participants were asked to rank three discrete health issues (drawn from issues identified in the broader study) in terms of priority: primary care and specialist access, transport to health services and drug and alcohol services. When ranking the three health issues, participants were asked to consider what the potential impact of addressing the issues was, who would benefit and why that group’s needs were most important to address. The purpose of this discussion was to understand the criteria underpinning their priority-settings decisions. Participants were then asked to individually rank their top three criteria, followed by a group discussion to justify ranking and allow individuals to revise their initial ranking informed by group reflections. The criteria identified were used in the statement rating activity in Step 3.

### Step 2: generation – formulating statements to represent identified health and healthcare issues

Data extracted from the community forum summaries and the survey responses were collated to provide a comprehensive set of data in response to the key question of interest “What do you think are the key health and healthcare issues in your community that need to be addressed?”. Three members of the research team then edited and synthesized this data set to ensure all statements were relevant and each idea was unique and only represented once and to improve the clarity and understanding of the statements if necessary. This process involved (1) deleting redundant statements (for example, random words as well as statements unable to be linked to the question of interest), (2) splitting compound statements, (3) identifying statements that represented the same ideas and selecting the statement that best captured the essence of the idea and, (4) where necessary, editing statements so that the essential meaning was clear. Throughout the editing and synthesizing process, we endeavoured to retain the original voice of the participants where possible. Discussion and editing continued until the members of the research team reached consensus. The final list of statements was cross-referenced to the original list to ensure all relevant ideas were represented.

### Step 3: structuring statements – statement sorting and rating

Participants completed two online concept mapping activities – sorting and rating the identified health and healthcare issues – using a project-specific page on the Concept System Global groupwisdom™ web-based application[28] between October and November 2020. The participants included community members, health professionals and academic researchers. Responses for each activity were confidential (participants did not know who else was participating or how they responded) but not anonymous (the research team could identify who provided what sorting and rating data). Only participants who completed the statement sorting activity were invited to complete the statement rating activity. Participants could access an 8-min instruction video [developed by two investigators (A.W.S. and A.D.)] on how to complete the online sorting and rating activities and navigate the web application (https://youtu.be/x7w9o9C-53E).

We aimed to recruit 15 participants per participant group (≥ 15 participants in three groups ≥ 45 participants). Previous research involving consensus methods, such as Delphi and concept mapping, have recommended 10–18 experts per participant group to ensure sufficient contributions, reliable outcomes and comparisons [19, 26, 27]. Community member and health professional participants in the brainstorming (Step 2) were eligible and invited to participate.

#### Statement sorting

The research team uploaded the edited and synthesized data set extracted from the community forum and survey responses into groupwisdom™. Participants were asked to (a) familiarize themselves with the statements (that is, healthcare and health issues) that emerged from the brainstorming (Step 2), (b) group similar or related statements together and (c) name each group they created on the basis of the topic that the statements had in common. Participants could put a single statement in a “group” if they thought the statement was unrelated to all other statements. Participants were asked not to group (a) statements on the basis of a value (for example, important or hard to do) and (b) unrelated statements together (for example, “miscellaneous” or “other”). They were informed that some participants created as few as 5 groups and others may create up to 15.

#### Statement rating

Participants rated each statement on a scale from 1 (nothing/not at all/none or hardly any) to 5 (a lot) for each of the three rating criteria identified through the stakeholder consultations (Step 2).

### Step 4: representation of statements

The participants’ sorting data were analysed using two-dimensional non-metric multidimensional scaling to locate each sorted statement as a separate point on a two-dimensional “point map”. The distances between the points on the cluster map is a proxy indicator of the degree of perceived similarity between the individual health and healthcare issues (that is, the statements grouped together by more participants are considered more “related” and generally located closer to each other on the map). We then used hierarchical cluster analysis to partition the statements on the point map into non-overlapping clusters of related statements (“cluster maps”) [28].

Kane and Trochim’s guidance to decide on the number of clusters was applied [18]. We focused on the statements that were merged as we reduced the number of clusters, with the aim of finding the cluster level that retained conceptually different clusters while merging conceptually similar clusters. Once the most appropriate cluster level was identified, if a statement on the map seemed to be a better conceptual fit in an adjacent cluster, we investigated it in more detail using the “spanning” feature of the groupwisdom™ software. This feature visually displays how frequently the statement was sorted with every other statement on the map. If supported by the quantitative data, statements were reassigned from the original cluster to the neighbouring cluster with which it seemed a better conceptual fit [29].

The bridging value for an individual statement is an indication of whether that statement was generally sorted with nearby statements (values close to 0) or with items located in other areas of the map (values closer to 1). Statements with lower bridging values are better indicators of the meaning of the part of the map they’re located in than statements with higher bridging values. The bridging value of a cluster is the average of the bridging values of the statements in the cluster and can be considered a proxy for the conceptual “tightness” of the cluster with a lower bridging value indicating a more stable and narrowly focused thematic content within the cluster.

We also calculated mean ratings for the values identified in the prioritization exercise for each statement which we used to create scatter plot “go-zones” [18].The resulting scatter plots were divided into four quadrants using the overall mean of each rating as the axes. A latent analysis which involved inductively studying the cluster map in combination with the go-zone map was used to identify higher level themes.

## Results

### Step 2: generation

#### Brainstorming

Three community forums were held from August to September 2019, and the survey was open from August to December 2019. There were 187 survey respondents – 117 community members (62.5%) and 70 health professionals (37.5%). Respondents’ characteristics are shown in Table 1. In all, 492 health and healthcare issue statements were extracted from the survey and community forum data. After statement synthesizing and editing, 72 unique local health and healthcare issues were retained for concept mapping participants to sort and rate.

**Table 1:** Demographic characteristics of survey respondents

	Community members (*N* = 117) *n* (%)	Health Professionals (*N* = 70) *n* (%)
Gender		
Female	86 (73.5)	58 (82.9)
Male	28 (23.9)	11 (15.7)
Prefer not to say	2 (1.7)	3 (4.3)
Other	1 (0.8)	0 (0.0)
Aboriginal and/or Torres Strait Islander		
Yes	3 (2.6)	2 (2.9)
No	110 (94.0)	66 (94.3)
Prefer not to say	2 (1.7)	1 (1.4)
Don’t know	2 (1.7)	1 (1.4)
Age group (years)		
18–30	21 (17.9)	12 (17.1)
31–40	15 (12.8)	20 (28.6)
41–50	25 (21.4)	14 (20.0)
51–60	27 (23.1)	18 (25.7)
> 60	29 (24.8)	6 (8.6)
Educational level		
Year 11/Form 5 or below	13 (11.1)	3 (4.3)
Year 12/Form 6	11 (9.4)	0 (0.0)
Certificate III or IV/Trade certificate	16 (13.7)	0 (0.0)
Diploma or advanced diploma	17 (14.5)	5 (7.1)
Bachelor or honours degree	21 (17.9)	14 (20.0)
Certificate or graduate diploma	8 (6.8)	14 (20.0)
Master’s or doctorate	11 (9.4)	17 (24.3)
Annual household income		
$19,999 or less	6 (5.1)	1 (1.4)
$20,000–$39,999	14 (12.0)	0 (0.0)
$40,000–$59,999	13 (11.1)	5 (7.1)
$60,000–$79,000	16 (13.7)	3 (4.3)
$80,000–$99.999	10 (8.5)	12 (17.1)
$100,000 or more	20 (17.1)	25 (35.7)
Self-rated health		
Poor	2 (1.7)	2 (2.9)
Fair	17 (14.5)	3 (4.3)
Good	49 (41.9)	14 (20.0)
Very good	31 (26.5)	36 (51.4)
Excellent	17 (14.5)	13 (18.6)

#### Identification of rating criteria

In all, 16 community members and 16 health professionals participated in six stakeholder consultation sessions. Two sessions (one community member group and one health professional group) were conducted face-to-face in one small rural town in February 2020. Four sessions (two community member and two health professional) were conducted June–July 2020 via the online platform Zoom [due to coronavirus disease 2019 (COVID-19) restrictions]. Community members and health professionals identified three key values that could be used to guide priority-setting: (1) equity – equal access for equal need; (2) health and social impact – number of people affected; and (3) capacity to address – capacity to implement an effective intervention.

### Step 3: structuring statements

A total of 51 participants took part in the statement sorting and/or rating activities between August and December 2020. The demographic characteristics for the participants in these activities are shown in Table 2.

**Table 2:** Demographic characteristics of concept mapping participants

	Sorting *n* (%)		Rating *n* (%)		Total *n* (%)
	*n* = 46	Capacity *n* = 47	Equity *n* = 43	Impact *n* = 42	*n* = 51
Gender					
Female	37 (80.4)	36 (76.6)	33 (76.7)	32 (76.2)	40 (78.4)
Male	9 (19.6)	11 (23.4)	10 (23.3)	10 (23.8)	11 (21.6)
Aboriginal and/or Torres Strait Islander					
Yes	1 (2.2)	1 (2.1)	(0.0)	(0.0)	1 (2.0)
No	45 (97.8)	46 (97.9)	43 (100.0)	42 (100.0)	50 (98.0)
Age group (years)					
18–30	5 (10.9)	6 (12.7)	5 (11.6)	5 (11.9)	6 (11.7)
31–40	11 (23.9)	12 (25.5)	11 (25.5)	10 (23.8)	12 (23.5)
41–50	12 (26.1)	13 (27.6)	11 (25.5)	11 (26.1)	14 (27.4)
51–60	11 (23.9)	9 (19.1)	9 (20.9)	9 (21.4)	12 (23.5)
> 60	7 (15.2)	7 (14.8)	7 (16.2)	7 (16.6)	7 (13.7)
Participant group					
Community member	12 (26.1)	13 (27.7)	13 (30.2)	13 (31.0)	14 (27.5)
Health professional	19 (41.3)	21 (44.6)	17 (39.6)	17 (40.4)	22 (43.1)
Researcher	15 (32.6)	13 (27.7)	13 (30.2)	12 (28.6)	15 (29.4)
Place of work (MMM*)					
Metropolitan (1)	5 (10.9)	4 (8.5)	4 (9.3)	3 (7.1)	5 (9.8)
Regional centre (2)	14 (30.4)	14 (29.8)	12 (27.9)	12 (28.6)	15 (29.4)
Large rural town (3)	5 (10.9)	7 (14.9)	5 (11.6)	5 (11.9)	7 (13.7)
Medium rural town (4)	4 (8.6)	3 (6.4)	3 (7.0)	3 (7.1)	4 (7.8)
Small rural town (5)	5 (10.9)	6 (12.8)	6 (14.0)	6 (14.3)	6 (11.8)
Unknown	13 (28.3)	13 (27.6)	13 (30.2)	13 (31.0)	14 (27.5)
Residential location (MMM)					
Metropolitan (1)	5 (10.9)	4 (8.5)	4 (9.3)	3 (7.1)	5 (9.8)
Regional centre (2)	25 (54.3)	25 (53.2)	23 (53.5)	23 (54.8)	27 (52.9)
Large rural town (3)	4 (8.7)	6 (12.8)	4 (9.3)	4 (9.5)	6 (11.8)
Medium rural town (4)	5 (10.9)	4 (8.5)	4 (9.3)	4 (9.5)	5 (9.8)
Small rural town (5)	7 (15.2)	8 (17.0)	8 (18.6)	8 (19.0)	8 (15.7)

*MMM = Modified Monash Model (30)

#### Statement sorting

The sorting data from 46 participants (12 community members, 19 health professionals and 15 academic researchers) was accepted for analysis (mean number of groups, 10.4; range, 3–25 groups). The sorting data for three participants was excluded from the analysis – one because they did not name the groups they created, and the research team could not see any meaning in the four groups they had created, and two because they grouped statements on the basis of a priority or value.

### Step 4: representation of statements

#### Cluster map

**Fig. 2:**
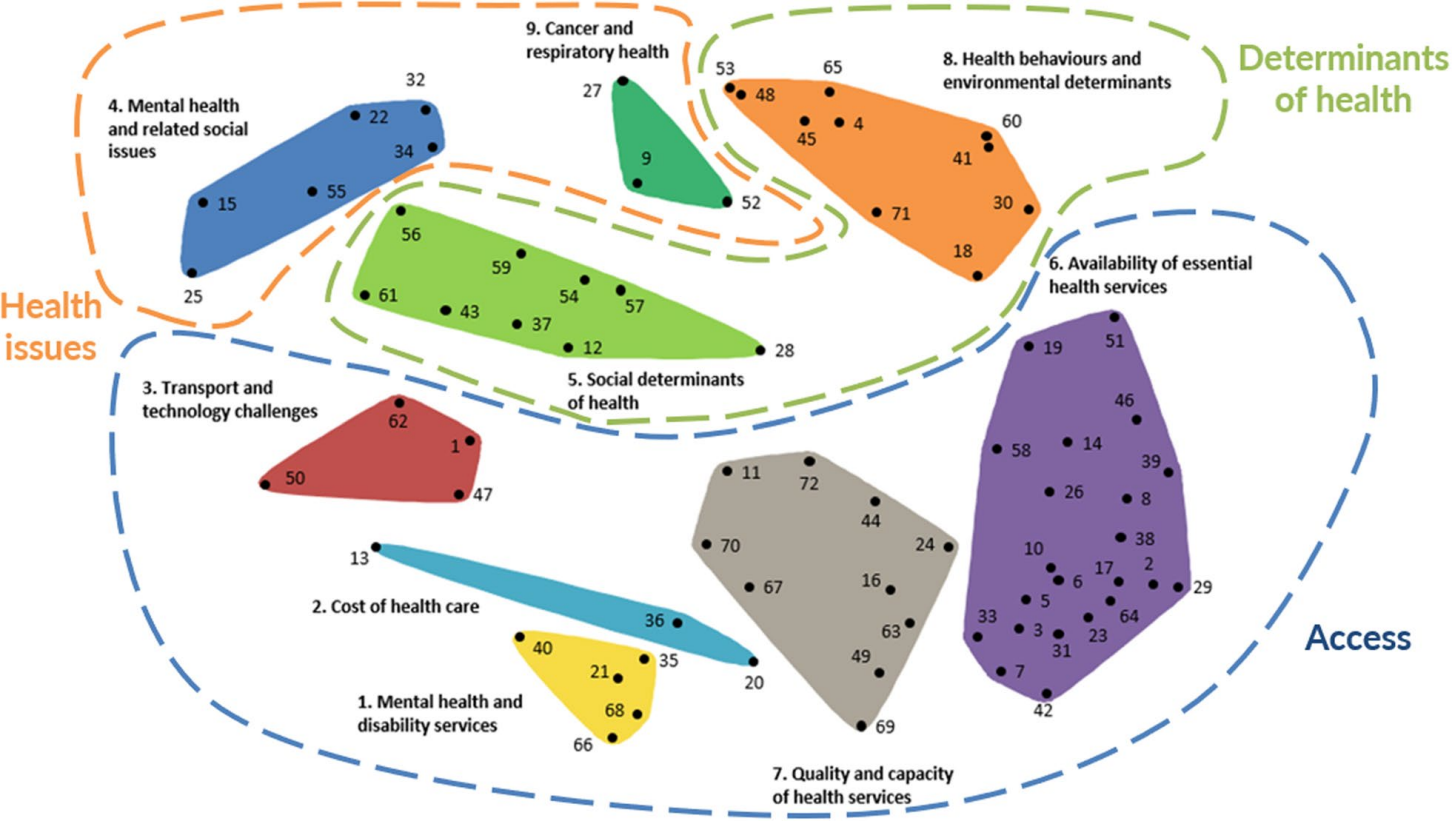
Nine-cluster map of local health and healthcare issues in Grampians region

A nine-cluster map (Fig. 2) was considered the most appropriate representation of the participants’ sorting data following multidimensional scale and hierarchical cluster analysis. The cluster names (decided by the research team informed by the names used by participants gave) and the associated health and healthcare issues contained in each cluster are shown in Table 3. The research team also grouped the clusters into three overarching domains of access, health issues and determinants of heath (Fig. 2).

**Table 3:** Cluster and associated health and healthcare issues

Cluster	Summary of cluster issues
Mental health and disability services	• The need for improved mental health service planning and coordination • Funding challenges related to the access and delivery of disability (NDIS) services • The need for evidence-based mental healthcare
Cost of healthcare	• Healthcare costs • Inequities in costs for people in rural areas
Transport and technology	• The impact of rurality on access to services and technology-based interventions
Mental health and related social issues	• The high rates and impact of mental health, substance abuse and social issues in rural communities
Social determinants of health	• Challenges vulnerable, disengaged and disadvantaged groups experience in navigating the healthcare system in terms of health literacy, advocacy and access to information support
Availability of essential health services	• The lack of timely access to essential healthcare services • Need for continuity of care • Challenges accessing specialist care • The need for a greater focus on preventative healthcare
Quality and capacity of health services	• The need to improve coordination between services and communication between healthcare providers and patients and between providers themselves, and the importance of providing accessible healthcare services and resources
Health behaviours and environmental determinants	• The need for health promotion and population health programs to address individual behaviour and system-related factors that contribute to poor health outcomes
Cancer and respiratory health	• The high rates of cancer and respiratory diseases in rural communities

NDIS, National Disability Insurance Scheme

The distances between the points on the cluster map (Fig. 2) are based on how frequently the statements were grouped together by participants. For example, statement #17 (*Long surgery waiting lists in public hospitals*) and #64 (*Long waiting times and high demand in emergency departments*) were considered so closely related that 41 of the 46 participants grouped them together. Consequently, they appear very close together on the map (in the “Health behaviours and environmental determinants” cluster). By contrast, statements #15 (There are high suicide rates in the region) and #29 (Specialist services are limited) were considered so unrelated that no participants grouped them together, and they are located on opposite sides of the map. The stress value of 0.2618 is below the average stress value (0.28) reported in a pooled-analysis of concept mapping studies [18], noting that lower stress values reflect a better fit between the participants’ original sorting data and the two-dimensional visual map.

#### Go-zones

**Fig. 3:**
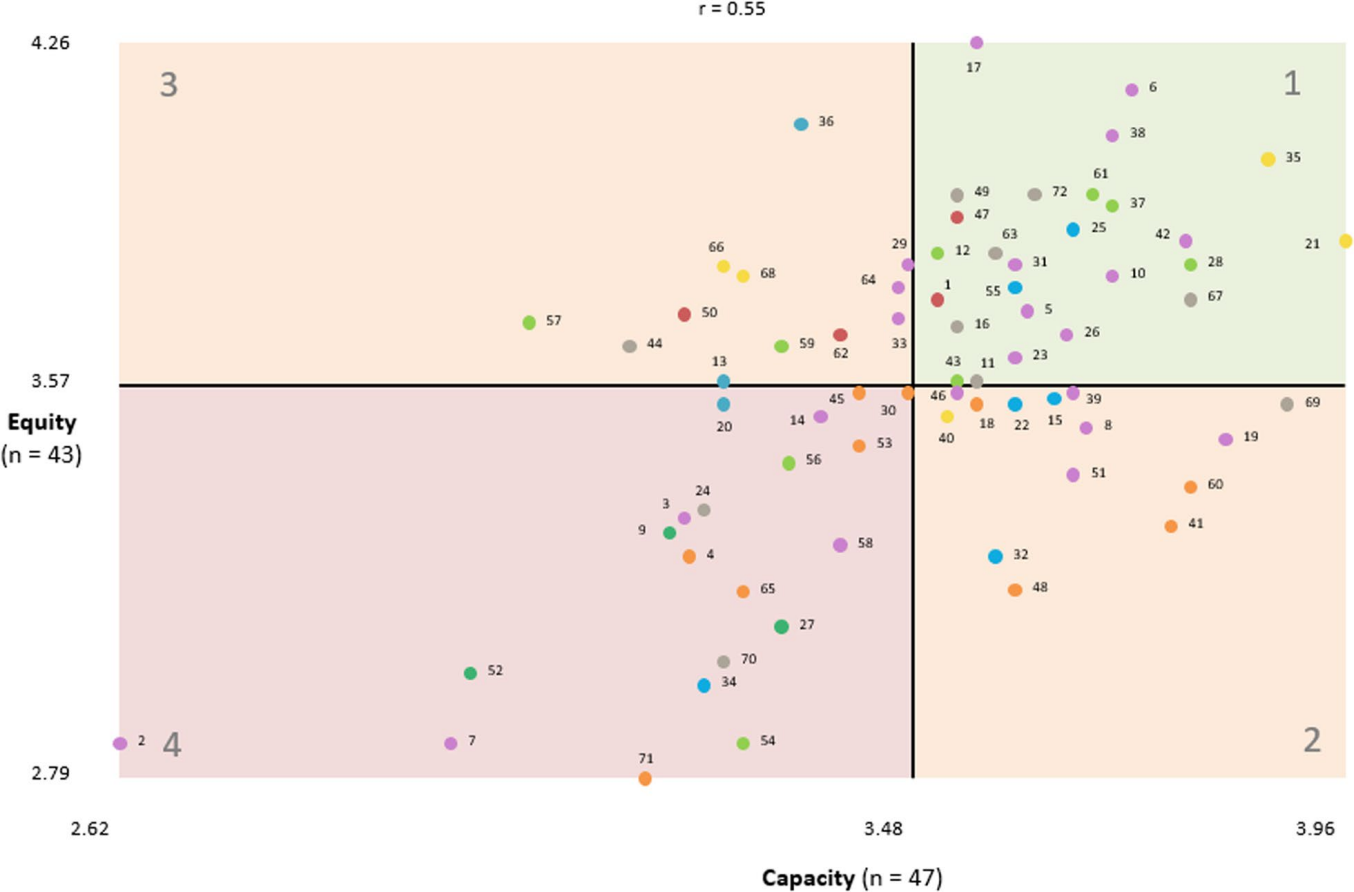
Go-zone graph of local health issues rated for equity versus capacity

**Fig. 4:**
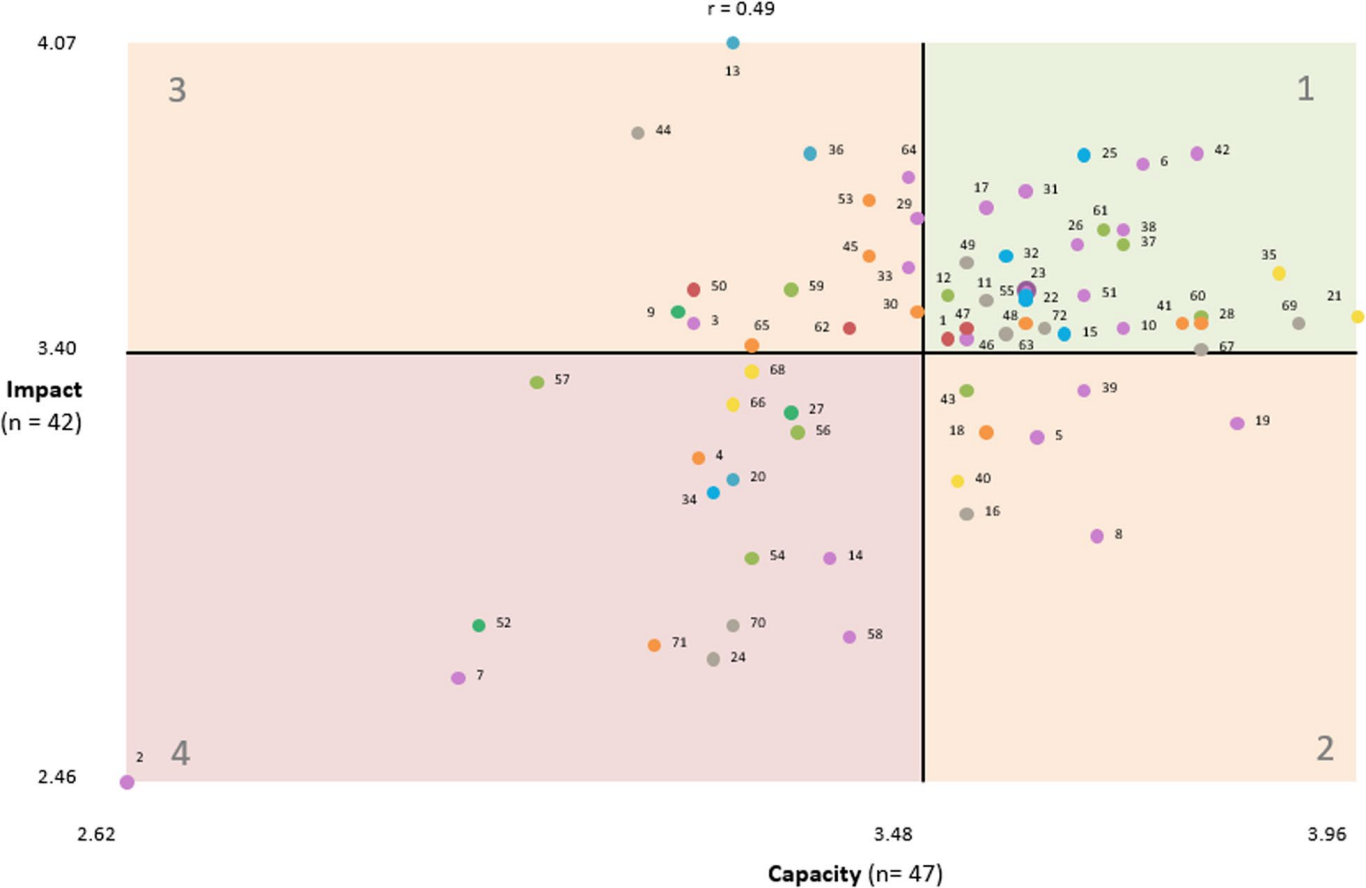
Go-zone graph of local health issues rated for impact versus capacity

**Fig. 5:**
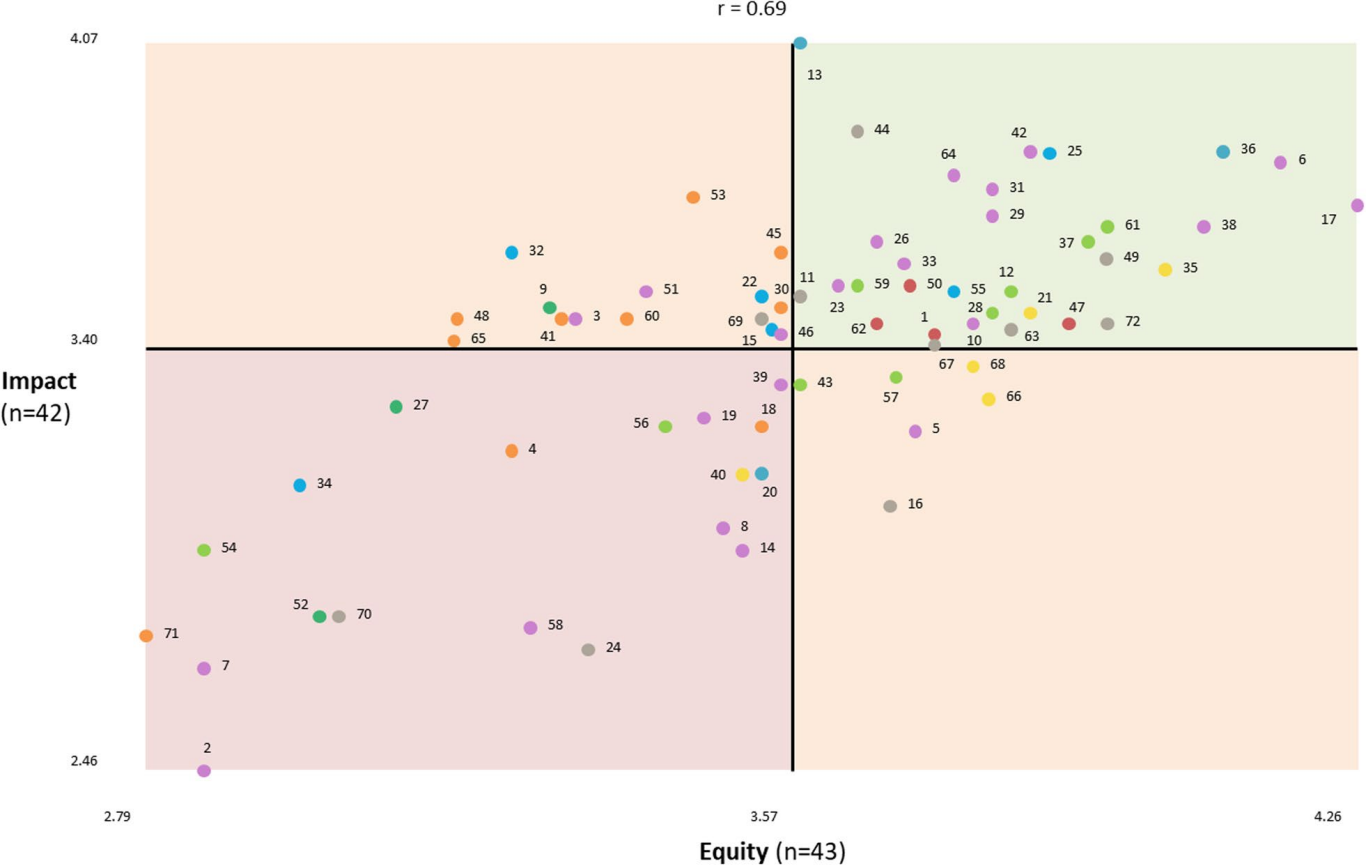
Go-zone graph of local health issues rated for impact versus equity

The go-zone graphs are shown for all 72 statements on the basis of their mean ratings for equity versus capacity (Fig. 3), impact versus capacity (Fig. 4) and impact versus equity (Fig. 5). The graphs are divided into quadrants above and below the overall mean of each rating, showing a “go-zone” quadrant of statements in the top right, which are above average on both scales. To aid interpretation of the go-zone, see Table 4 for the details of each statement including its mean ratings and go-zone graph quadrant.

**Table 4:** Health and healthcare issues in the community that need to be addressed with mean impact, capacity and equity ratings

Cluster Statement number – health/healthcare issue		Mean rating				Go-zone quadrant^+^		
		Bridging score^**a**^	Capacity^**b**^	Equity^c^	Impact*	E versus C	I versus C	I versus E
1. Mental health and disability services		0.65	3.59	3.80	3.36			
35	Mental health services are fragmented.	0.62	3.87	4.02	3.57	1	1	1
21	Poor discharge planning and follow-up for mental health.	0.64	3.96	3.86	3.48	1	1	1
68	Funding models for health programs [for example, National Disability Insurance Scheme(NDIS)] restrict the ability of the health service to meet the needs of all community stakeholders.	0.53	3.30	3.79	3.36	3	4	2
66	Funding models for NDIS are challenging for service providers.	0.54	3.28	3.81	3.29	3	4	2
40	Inappropriate prescribing of medications for mental health.	0.90	3.52	3.51	3.12	2	2	4
2. Cost of healthcare		0.57	3.31	3.74	3.68			
36	Expensive and at times unreasonable cost of healthcare.	0.43	3.36	4.09	3.83	3	3	1
13	Private health insurance is expensive.	0.89	3.28	3.58	4.07	3	3	1
20	People in some rural areas have to pay for emergency care.	0.40	3.28	3.53	3.12	4	4	4
3. Transport and technology challenges		0.71	3.42	3.76	3.47			
47	Limited transport options if someone is temporarily or permanently unable to get themselves around.	0.64	3.53	3.91	3.45	1	1	1
1	Technology-based interventions are hard to apply to the community where people do not have internet.	0.58	3.51	3.74	3.43	1	1	1
62	Lack of public transport in the region.	0.69	3.40	3.67	3.45	3	3	1
50	High travel costs.	0.91	3.23	3.71	3.54	3	3	1
4. Mental health and related social issues		0.62	3.55	3.49	3.50			
25	Increased anxiety/mental health in young people in the region.	1.00	3.66	3.88	3.83	1	1	1
55	High incidence of family violence in the region.	0.49	3.60	3.77	3.52	1	1	1
22	Substance abuse and overuse is high in the region.	0.52	3.60	3.53	3.51	2	1	3
15	There are high suicide rates in the region.	0.89	3.64	3.55	3.44	2	1	3
32	Alcohol misuse is high in the region.	0.40	3.57	3.23	3.61	2	1	3
34	There is a high incidence of gambling in the region.	0.42	3.26	2.98	3.10	4	4	4
5. Social determinants of health		0.46	3.47	3.64	3.41			
61	The social isolation and disconnection of vulnerable groups in the community.	0.57	3.68	3.95	3.67	1	1	1
37	People are unaware or unable to advocate for their healthcare needs.	0.56	3.70	3.93	3.63	1	1	1
28	Lack of health information and care for disadvantaged families.	0.43	3.79	3.81	3.48	1	1	1
12	Health messages are heard and understood but cannot always be acted upon due to financial pressures.	0.50	3.51	3.84	3.52	1	1	1
59	There are pockets of poverty and disadvantage in the region.	0.35	3.34	3.65	3.54	3	3	1
43	Low levels of technology literacy (for example, using computers) in vulnerable groups in the region.	0.48	3.53	3.58	3.32	1	2	2
57	Housing is either unavailable or unaffordable in the region.	0.38	3.06	3.70	3.33	3	2	2
56	Racism and discrimination in the region.	0.44	3.35	3.42	3.23	4	4	4
54	Limited “things to do” for all age groups in the region.	0.42	3.30	2.86	2.95	4	4	4
6. Availability of essential health services		0.20	3.51	3.62	3.39			
6	Long waiting lists for affordable dental services.	0.09	3.72	4.16	3.81	1	1	1
17	Long surgery waiting lists in public hospitals.	0.04	3.55	4.26	3.71	1	1	1
42	Difficult to recruit and retain medical staff in the region.	0.14	3.78	3.86	3.83	1	1	1
38	There are not many local mental health services.	0.39	3.70	4.07	3.67	1	1	1
31	GP shortages and inability to see a GP in a timely and affordable manner.	0.03	3.60	3.81	3.75	1	1	1
64	Long waiting times and high demand in emergency departments.	0.02	3.47	3.77	3.78	3	3	1
29	Specialist services are limited.	0.12	3.48	3.81	3.69	3	3	1
26	Lack of resources for chronic disease management.	0.19	3.65	3.67	3.63	1	1	1
10	Lack of support services for carers, for example, disability and dementia.	0.05	3.70	3.79	3.45	1	1	1
23	Lack of after-hours services increases demand on health services.	0.00	3.60	3.63	3.54	1	1	1
33	Limited opportunity to have ongoing care from the same GP.	0.04	3.47	3.71	3.59	3	3	1
51	Lack of early intervention to help people with healthy behaviours, for example, exercise and healthy eating.	0.60	3.66	3.40	3.52	2	1	3
5	Lack of timely access to allied healthcare for children.	0.02	3.61	3.72	3.21	1	2	2
46	Lack of services to manage obesity in the region.	0.42	3.53	3.56	3.43	2	1	3
19	Underuse of prevention and non-surgical management of musculoskeletal conditions (for example, OA).	0.58	3.83	3.47	3.24	2	2	4
39	Lack of alcohol and drug support services.	0.55	3.66	3.56	3.32	2	2	4
8	Limited sexual health services for young people.	0.21	3.67	3.49	3.00	2	2	4
3	Lack of medical practitioner choices in public health services.	0.00	3.23	3.31	3.46	4	3	3
14	Lack of services for people with eating disorders in the region.	0.35	3.38	3.51	2.95	4	4	4
58	There are minimal local support services for LGBTIQ community.	0.33	3.40	3.26	2.78	4	4	4
7	Generally poor quality of medical professionals in the region.	0.10	2.98	2.86	2.69	4	4	4
2	Local hospitals do not birth babies.	0.13	2.62	2.86	2.46	4	4	4
7. Quality and capacity of health services		0.30	3.52	3.63	3.33			
49	Difficult to get home care support packages.	0.21	3.53	3.95	3.60	1	1	1
72	Limited access and inclusion for people with a disability.	0.38	3.62	3.95	3.45	1	1	1
67	Lack of and poor coordination of support services in the community.	0.37	3.79	3.74	3.40	1	1	1
69	There is minimal communication among and coordination of health services.	0.32	3.89	3.53	3.46	2	1	3
63	Lack of appropriate and quality residential aged care facilities.	0.14	3.57	3.84	3.44	1	1	1
44	Distance to services limits access.	0.34	3.17	3.65	3.88	3	3	1
11	Chronic pain management, including use of opiates, is challenging for patients, families, allied health and doctors.	0.50	3.55	3.58	3.51	1	1	1
16	Health services are not built for less mobile people.	0.14	3.53	3.69	3.05	1	2	2
24	Specialized medical supplies, for example, catheters and wound dressings, can be hard to get.	0.12	3.26	3.33	2.73	4	4	4
70	Lack of trust in health professional and the health system.	0.49	3.28	3.02	2.80	4	4	4
8. Health behaviours and environmental determinants		0.38	3.48	3.31	3.38			
60	Lack education on diet and healthy food preparation.	0.38	3.79	3.37	3.46	2	1	3
53	Obesity has a big personal and public cost.	0.30	3.43	3.45	3.73	4	3	3
45	Processed and fast food is cheaper and easier to get than nutritious healthy food.	0.28	3.43	3.56	3.61	4	3	3
41	Lack of focus on healthy living/lifestyle in the region.	0.38	3.77	3.29	3.46	2	1	3
30	People do not understand health messages or do not think they are relevant.	0.60	3.48	3.56	3.49	4	2	3
18	People delay getting tested for cancer symptoms.	0.57	3.55	3.53	3.23	2	2	4
48	Low physical activity levels in the region.	0.26	3.60	3.17	3.46	2	1	3
65	Public attitudes towards obesity mean overweight people are less likely to engage in healthy behaviours.	0.29	3.30	3.16	3.41	4	3	3
4	Poor food quality and choices in rural areas.	0.28	3.24	3.23	3.17	4	4	4
71	Limited recreational and sporting facilities and opportunities in the region.	0.51	3.19	2.79	2.76	4	4	4
9. Cancer and respiratory health		0.43	3.19	3.12	3.19			
9	High incidence of cancer in the region.	0.44	3.22	3.28	3.49	4	3	3
27	High rates of smoking in the region.	0.37	3.34	3.09	3.27	4	4	4
52	High incidence of respiratory diseases among farmers.	0.47	3.00	3.00	2.80	4	4	4
All statements (Grand mean)			3.48	3.57	3.40			

+ 1 = above the grand mean on both scales; 2 = above the grand mean on the *x*-axis scale and below the grand mean on the *y*-axis scale; 3 = below the grand mean on the *x*-axis scale and above the grand mean on the *y*-axis scale; and 4 = below the grand mean on both scales. GP, general practitioner; OA, osteoarthritis

^+^Go-zone graph is divided into four quadrants above and below the overall mean of each rating: quadrant 1 includes statements which are above average on both ratings; quadrant 4 includes statement which are below average on both ratings; and quadrants 2 and 3 include statements which are above average on one rating and below average on the other rating

^a^Values closer to 0 represent clusters with more coherent contents or anchoring statements with stronger links to nearby statements. Values closer to 1 represent clusters with less coherent content or bridging statements with stronger links to statements in other sections of the map

^b^How much can be done about this issue in the Grampians region? (1 = nothing; 5 = a lot)

^c^How much would addressing this issue ensure those with equal health need have equal access to care? (1 = not at all; 5 = a lot)

*How many people in the Grampians region does this issue impact? (1 = none or hardly any; 5 = a lot)

#### Prioritization

Issue statements were defined as actionable priorities if they were in the go-zone quadrant (quadrant 1, Figs. 3, 4, 5) for all three go-zone graphs. For example, statement #35 “mental health services are fragmented” rated above average on all three criteria – capacity to address (3.87 out of 5), health equity (4.02) and impact (3.57). In addition, statements that were in quadrant 1 for at least one of the go-zone graphs and in the top left (quadrant 3) for the other two go-zone graphs were also considered potential priorities. For example, statement #36 “expensive and at times unreasonable cost of healthcare” was in quadrant 1 for the go-zone graph of impact versus equity, and in quadrant 3 for the go-zone graphs equity versus capacity and impact versus capacity. This indicates that this issue is important in terms of equity and impact, but participants considered this more difficult to address than some of the other issues.

A worked example of the analysis is shown in the supplementary file.

## Discussion

This study has identified actionable health and healthcare priorities for healthcare delivery in rural communities in the Grampians region. We have demonstrated the feasibility of undertaking consensus-based priority-setting activities for large geographical regions with disparate and heterogeneous communities and stakeholder groups. Using in-depth community and health professional stakeholder engagement across the region and a group concept mapping process, nine action areas were identified: (1) mental health and disability services, (2) cost of healthcare, (3) transport and technology challenges, (4) mental health and related social issues, (5) social determinants of health, (6) availability of essential health services, (7) quality and capacity of health services, (8) health behaviours and environmental determinants and (9) cancer and respiratory health. These areas can be conceptualized in three overarching domains: healthcare access, health issues and determinants of health. The following discussion focuses on the issues that were identified as priorities.

### Healthcare access

The majority of issues that were identified as actionable priorities were related to Penchansky and Thomas’ dimensions of access [30] – acceptability, accommodation, availability and accessibility. In addition, the fifth dimension of access “affordability” was identified as important in terms of health equity and impact; however, affordability issues were considered more difficult to address.

Our study identified a number of issues associated with acceptability, that is, patients’ perceptions of a provider’s level of service and competency [31]. Continuity of care, in terms of ongoing care from the same general practitioner or health provider, was identified as a priority issue in terms of impact and equity but not in terms of capacity to address. This is consistent with previous research examining rural healthcare access that found that people in rural areas were more concerned about being able to use general practitioner (GP) services that they prefer, than with distance to the service [32, 33]. It may also reflect an awareness of generally declining continuity of care for regular users of general practice in Australia [34]. These issues are compounded in rural communities because of the limited choice of providers and the high proportion of rural and remote primary healthcare providers who have insufficient orientation and support [35].

Participants described several challenges regarding the accommodation (or adequacy) of healthcare services – consumers’ “ability to contact, gain entry and navigate the system at times of need” [33]. The lack of affordable after-hours services was identified as an issue in rural areas. A recent review of health services accessibility for older Australians reported that many GP practices in rural areas are at capacity and that after-hours appointments were limited or not available, with flow-on effects as GPs are gatekeepers to other healthcare providers [36]. Previous research comparing the extent to which access barriers were experienced in Australia with other countries found Australians faced more difficulties with after-hours access than half of the comparator countries, including Canada, UK and USA [37]. Long waiting lists for dental services and surgery and a lack of specialized services for mental health were also identified as priorities in our study.

Participants raised concerns about the lack of coordination of services and challenges navigating the healthcare system, particularly for people with mental health issues, disability and low health literacy. Healthcare services are often fragmented, and there is inadequate oversight of the care of individual patients [38]. Poor care coordination can lead to poor patient management, medical errors and higher costs [39].

The differences in care integration and coordination experience between rural and metropolitan patients are well recognized. The likelihood that a patient’s usual GP or place of care will be informed of their follow-up needs after they have seen a health professional, visited a specialist or have been admitted to hospital reduces as remoteness increases [40]. To address this disparity, the Australian Government has developed the National Strategic Framework for Rural and Remote Health [41] to improve the coordination of care between rural healthcare providers.

Problems with availability in terms of inadequate provision of the volume and types of services to meet the healthcare needs of rural communities were identified in our study (for example, the insufficient supply of general practitioners, dentists and other healthcare providers). State and national governments have long sought to address workforce maldistribution in rural Australia. However, as with many countries, the situation has worsened since this study was undertaken. Effective solutions remain elusive, and the problem intractable, despite long-term investment [35]. The chronic workforce shortages across many health professions, which worsen as remoteness increases [35], are compounded by the higher levels of disease and injury for people in rural areas compared with those in metropolitan settings [12]. Availability issues have a significant impact on rural and remote populations, and there is a pressing need to develop new health workforce models that meet the needs of rural communities.

Participants in our study raised a number of challenges related to poor accessibility of services and technology-based interventions. Issues included lack of public transport in rural areas, high travel costs and ineffectiveness of technology-based interventions for people in rural areas with poor access to reliable internet coverage. Physical access to health services is a persistent challenge in Australian rural areas, with the vast distances, uneven population distribution and workforce maldistribution. The concomitant inequitable distribution of health services [42] is problematic, as proximity to healthcare services is a strong predictor of health disparity [43]. Services that are perceived as accessible by patients are associated with stronger relationships with providers and greater service utilization, leading to better overall health [31].

Our study identified issues related to affordability in terms of the costs for the consumer, including direct medical costs (for example, out-of-pocket payments) and indirect costs, such as costs of travelling to get care. Participants raised concerns about the costs of healthcare and the inequity in costs for people in rural areas. For example, some people living in rural areas were required to pay for emergency care, whereas people living in cities had access to publicly funded emergency care. There is increasing evidence of the substantial impact of out-of-pocket costs and the flow-on effects on healthcare access and health outcomes for people in rural areas [44]. A recent study showed that 1 in 4 Australians with a chronic health condition do not seek care due to the cost [45]. Given that people living in rural areas are often poorer, have to travel further to get healthcare, face higher out-of-pocket costs and have poorer health status compared with those living in metropolitan areas, [12] affordability of healthcare is critical.

### Health issues and health determinants

Concerns were raised in our study about the high rates and impact of mental health, substance abuse and social issues in these rural communities. Related to this, there was considerable priority afforded to the health issues and health determinants for vulnerable communities, including those experiencing housing insecurity, poor health literacy, poverty and inadequate access to internet and transport. Consistent with findings from the recent Victorian Royal Commissions (the highest form of public inquiry in Australia [46] for matters of public importance) into family violence [47] and mental health [48], high priority issues in our study included the increase in anxiety and mental health problems for younger people, and the high rates of domestic violence in the region. The system-wide mental health workforce shortages are magnified in rural areas, with young people in these communities facing “a number of challenges when accessing treatment, care and support, among them stigma and a lack of local services” [48]. The recent Royal Commission into Victoria’s mental health system highlights the importance of investing in the mental health and well-being of young people because of the potential adverse effects into adulthood [48]. Similarly, the Royal Commission into Family Violence identified multiple factors that influence how family violence is experienced in rural communities including geographic and social isolation, the perpetrator’s position within the community and the inability to maintain privacy, and economic disadvantage leading to victims’ economic dependence on their partner and family [47]. The recommendations from both Royal Commissions represent an opportunity for system-wide change to ensure high quality mental health and family violence services that meet community needs.

It is important to note that publicity arising from these Royal Commissions may have had a direct impact on the high priority given to mental health and certain social issues, as opposed other health issues such as dementia, cardiovascular disease and diabetes, which also contribute substantially to the Australian population health burden. Revelations about shortcomings in care delivery might resonate strongly in the region, given the observed importance of equity to the community. Royal Commissions are perceived as highly independent, are seen to “speak truth to power” and significantly influence policy [49]. However, they are often a measure of last resort. Therefore, our findings might reflect that these areas have been sorely neglected, and that consumers, advocates and health and social care professions may perceive a heightened a sense of agency to advocate for their causes through processes such as ours. Our consensus building approach at a regional level might be a useful tool to identify priority local issues from within the broad range of recommendations emanating from Commission reports.

### Patient and public involvement

It is well recognized that rural and urban communities differ, not only in the health and healthcare challenges faced but also the barriers to participation in healthcare decision-making, such as geographical isolation, lack of transportation, confidentiality and culture [50]. These differences highlight the need to ensure that engagement initiatives are fit for purpose in rural communities [51]. The use of the structured concept mapping process in this study enabled multi-level, meaningful rural community engagement. The brainstorming activity enabled a diverse range of stakeholders, including patients and the public, to contribute their local knowledge. The strategies used in the brainstorming, in particular the community forums and stakeholder consultations brought the research to the communities where people live and work, contributing to genuine engagement with rural community members and health professionals.

The use of the online platform enabled participants from many geographical locations to participate at a time that suited them and limited the influence of group-think and power imbalances between respondents. Consensus methods, such as Delphi, can be resource intensive, costly and logistically challenging. The online platform reduced the demand for resources, such as staff and participant time and travel.

Community and stakeholder engagement is multi-level and occurs on a continuum, as outlined in the International Association for Public Participation (IAP2) Public Participation Spectrum [52]. The limited evidence generally around mechanisms for health priority-setting with communities, which is amplified in rural areas, creates a void in terms of recommendations and clear frameworks for engagement [16]. This in turn heightens the risk of tokenistic approaches to community engagement. The output of our study consequently provides a valuable benchmark for future rural priority-setting research in terms of feasibility. While this concept mapping approach was a feasible mechanism for informing, consulting and involving community members, there is the need for further work that involves collaborating with and empowering patients and the public in these communities [52]. Process and outcome evaluations of the concept mapping approach were not within the scope or funding of this study. Therefore, there is a need for further research to understand the experiences and perceptions of participants, community members in particular, in regard to concept mapping as a participatory approach.

### Strengths and limitations

We had broad stakeholder involvement (multisectoral and multidisciplinary), which was beneficial for several reasons: different groups of stakeholders prioritize health and healthcare issues differently [53]; it helps ensure health and healthcare issues are not overlooked; broad participation helps ensure the prioritized issues meet the needs of those that will implement and those that will benefit, and increases the chances of addressing the priorities.

A broad range of responses, beyond specific individualized health issues, provides a deep understanding of the health and healthcare issues of communities in the region and indicates the varied interpretation of the prompt question “What do you think are the key health and healthcare issues in your community that need to be addressed?”. The process of identifying and prioritizing enabled rural community members, health professionals working in rural public health services and academics to have a voice regarding what are important health and healthcare issues to them. There was strong engagement with this work, demonstrated by the high retention rate of participants for the online concept mapping sorting and rating activities – 46 of the 51 participants who agreed to participate completed both activities.

Similar to other qualitative methods, there are a number of methodological limitations to concept mapping including issues around the reliability, validity and generalizability of the findings due to the small sample size and non-random sampling [54]. There were substantially more female community member participants than males. Also, a very low proportion of community member who participated, as well as low number of health professionals and academics, identified as Aboriginal and/or Torres Strait Islander. Given the recent endorsement of the Victorian Aboriginal Research Accord [55], further culturally appropriate research is needed to identify health and healthcare priorities that are meaningful for Aboriginal and/or Torres Strait Islander peoples. The time commitment for participation and the use of the online platform may have resulted in participants that were not representative of the communities. While this study involved a range of community members and health professionals from five rural communities in Western Victoria, issues related to inclusion, representation and the types of knowledge that are considered legitimate exist. The finding of this study may not be representative of those communities and may not be generalizable to other rural communities.

Four out of the six stakeholder consultations to identify the rating criteria and the online sorting and rating activities were conducted during the COVID-19 pandemic. While this did not impact on participation (similar numbers in each session), the pandemic may have influenced the values participants used to identify the rating criteria and how they rated each issue on the basis of these criteria.

In addition, although the research team followed standard and well-defined concept mapping procedures [18], they still used their subjective judgement and professional expertise to synthesize and edit the number, boundaries and names of clusters for the cluster map.

### Implications

Implications for policy: While the areas of cost of healthcare and mental health and disability services rated highly in terms of equity, capacity to address and impact, it is important to note that many of the issues, clusters and domains are interrelated and not mutually exclusive. These findings highlight the complexity of need and that it would be inappropriate to try to address individual issues in isolation.

Implications for practice: When addressing healthcare access, health services and healthcare providers should consider that people perceive their access to health services to be more than just the number of providers available. Therefore, those designing and delivering services need to consider the factors likely to affect consumers’ perceived affordability, acceptability and availability of care. Providers should also consider patient need for increased support with care coordination and access – difficulties with navigating the system and lack of timely access to appropriate local services appear common.

Implications for future research: Findings provide a mandate for priority healthcare innovation and research topics focusing on access issues and vulnerable patient groups in this rural setting. It would be useful to explore the universality of different priority issues across different rural areas to better determine the need for local versus macro-level initiatives. Further work is needed to understand rural consumer perspectives about what makes a service “accessible” and to develop “researchable” topics that address the most important needs of people in rural communities.

### Utilization

A summary of study findings was shared with participants and the health organization. These findings have led to the funding of a collaborative research centre, the Centre for Australian Research into Access (https://greateraccessforall.com/), which focuses on healthcare access and will provide information on the detailed needs of the communities and inform decision-makers on resource allocation.

## Conclusions

Identifying the most important health and healthcare issues for those that will use or deliver the services is a key step in optimizing healthcare. Our study used a structured approach to gather and evaluate multiple perspectives and elicit a diverse range of stakeholders’ health and healthcare priorities. This approach aligns with the growing recognition of the value of involving end-users in research and healthcare priority-setting, particularly in underserved areas. Our findings highlight that issues related to access, such as the inequities in healthcare costs, the perceived lack of quality and availability of services, particularly in mental health and disability, are important to people living and working in rural communities. It has also highlighted the complexity of need and the importance of a multi-strategy systems approach to addressing these issues in rural areas. The identified health and healthcare priorities are a valuable resource and have informed local health research and policy decisions. Decision-makers can use this information to allocate resources effectively and propose targeted solutions that address the specific needs of these rural communities.

### Supplementary Information

Additional file 1.

## Data Availability

The corresponding can be contacted regarding availability of data and materials.
