# Supplementary material for: Identifying health and healthcare priorities in rural areas: A concept mapping study informed by consumers, health professionals and researchers
Source: Health Res Policy Syst. 2024 Aug 13;22:105. doi: 10.1186/s12961-024-01163-1 (PMC11320881; doi:10.1186/s12961-024-01163-1)

**ANALYSIS – WORKED EXAMPLE**

**Brainstorming**

72 unique health and healthcare issue statements identified.

**Identification of rating criteria**

Three rating criteria were identified:

- Capacity to address: how much can be done about this issue in the Grampians region?
- Equity: how much would addressing this issue ensure those with equal health need have equal access to care?
- Impact: how many people in the Grampians region does this issue impact?

Note: we have used two of the rating criteria for this worked example

**Sorting**

Participants sorted the 72 unique statements into groups of similar or related issues.

- 46 participants created a mean number of 10.4 groups (range: 3–25 groups)

Based on multidimensional scaling analysis of the participants’ sorting, each statement is located as a point on a ‘point map.’

**Point map**

All 72 statements are shown as points and their location on the map is determined by how often they were sorted into a group with another statement by the participants.

For example,

- 37 of the 43 participants whose sorting data was included in the multidimensional scaling analysis sorted Statement 35 “Mental health services are fragmented” and Statement 21 “Poor discharge planning and follow up for mental health” into the same group. Therefore, these two statements are located close together on the point map.
- None of the 43 participants sorted Statements 35 and 27 “High rates of smoking in the region” into the same group. Therefore, they are situated far apart on the map.


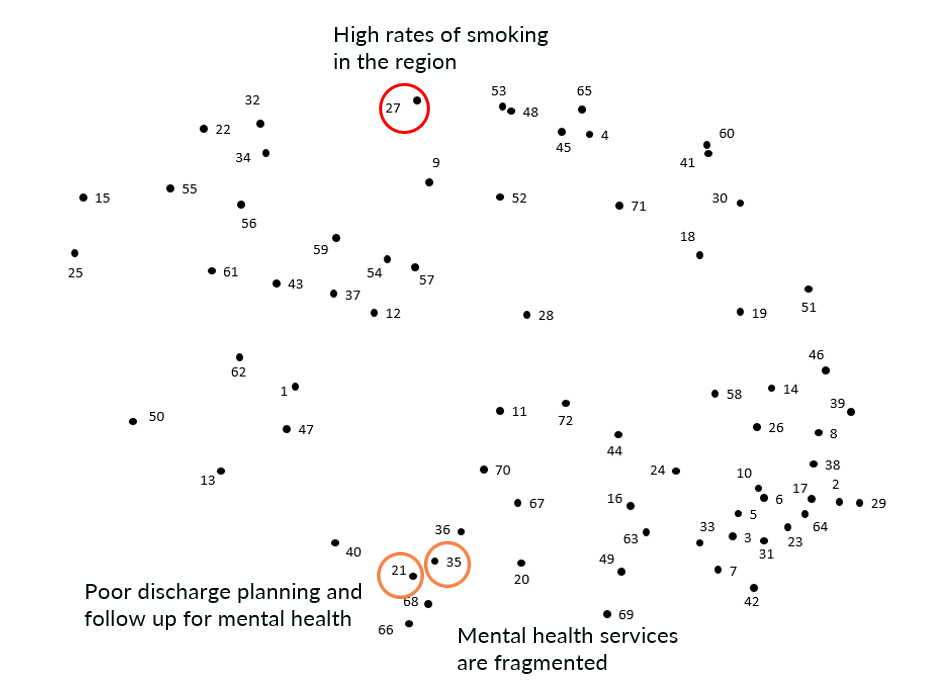


**Cluster map**

Hierarchical cluster analysis was used to group individual statements on the point map into clusters of statements of related ideas. This cluster map shows a 9-cluster solution.

Note: the points stay in the same position


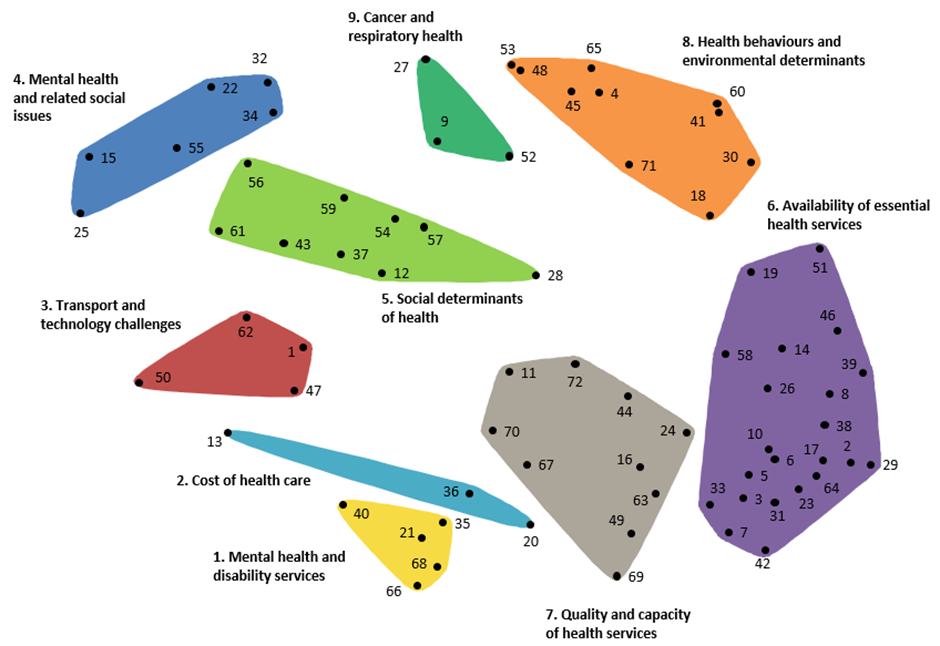

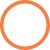

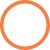


The table shows the three statements, their cluster name, and the rating (out of 5) that statement was given for two priority setting criteria.

| Cluster | Health care issue (statement number) | Capacity to address¹ | Equity² |
| --- | --- | --- | --- |
| Mental health and disability services | Mental health services are fragmented (35) | 3.87 | 4.02 |
| Mental health and disability services | Poor discharge planning and follow up for mental health (21) | 3.96 | 3.86 |
| Cancer and respiratory health | High rates of smoking in the region (27) | 3.34 | 3.09 |
| ¹How much can be done about this issue in the Grampians region?; ²How much would addressing this issue ensure those with equal health need have equal access to care? | | | |

**Go-zone graph**

The mean ratings for each statement for the two priority setting criteria — *equity* and *capacity to address* — are mapped in the ‘go-zone’ graph below.

All the statements are colour coded according to their cluster group.

Statements in the top right quadrant were rated above the grand mean for both *equity* and *capacity to address* i.e. participants perceived that there was something that could be done about the issue and if it was done it would improve access to care for those that need it.

The go-zone graph helps us identify which issues to prioritise:

- Statement 35 “Mental health services are fragmented” and Statement 21 “Poor discharge planning and follow up for mental health” are both in quadrant 1 of the go-zone (i.e. rated as above the grand mean for both equity and capacity to address). Therefore, they should be considered as a potential priority to address.
- Statements 27 “High rates of smoking in the region” is rated below the grand mean for both equity and capacity to address and is located in quadrant 4 of the go-zone graph. Consequently, it should be considered a low priority for attention or intervention.


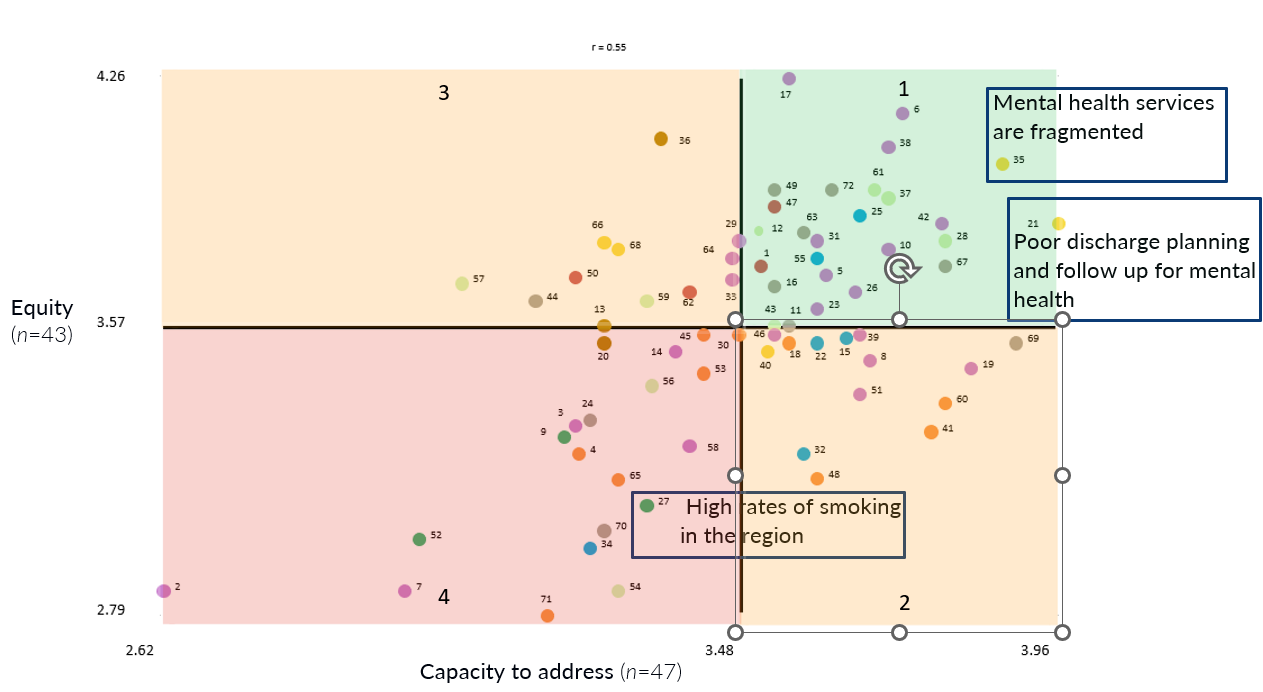

Supplement: Supplementary file 1 — Additional file 1. [file 12961_2024_1163_MOESM1_ESM.docx]
